# Drug Repositioning in Friedreich Ataxia

**DOI:** 10.3389/fnins.2022.814445

**Published:** 2022-02-09

**Authors:** Alessandra Rufini, Florence Malisan, Ivano Condò, Roberto Testi

**Affiliations:** ^1^Department of Biomedicine and Prevention, University of Rome “Tor Vergata”, Rome, Italy; ^2^Fratagene Therapeutics, Rome, Italy; ^3^Saint Camillus International University of Health and Medical Sciences, Rome, Italy

**Keywords:** friedreich ataxia, drug development, frataxin, drug repositioning, therapeutics

## Abstract

Friedreich ataxia is a rare neurodegenerative disorder caused by insufficient levels of the essential mitochondrial protein frataxin. It is a severely debilitating disease that significantly impacts the quality of life of affected patients and reduces their life expectancy, however, an adequate cure is not yet available for patients. Frataxin function, although not thoroughly elucidated, is associated with assembly of iron-sulfur cluster and iron metabolism, therefore insufficient frataxin levels lead to reduced activity of many mitochondrial enzymes involved in the electron transport chain, impaired mitochondrial metabolism, reduced ATP production and inefficient anti-oxidant response. As a consequence, neurons progressively die and patients progressively lose their ability to coordinate movement and perform daily activities. Therapeutic strategies aim at restoring sufficient frataxin levels or at correcting some of the downstream consequences of frataxin deficiency. However, the classical pathways of drug discovery are challenging, require a significant amount of resources and time to reach the final approval, and present a high failure rate. Drug repositioning represents a viable alternative to boost the identification of a therapy, particularly for rare diseases where resources are often limited. In this review we will describe recent efforts aimed at the identification of a therapy for Friedreich ataxia through drug repositioning, and discuss the limitation of such strategies.

## Introduction

Friedreich ataxia (FRDA, OMIM#229300) is a rare genetic disorder caused by insufficient levels of the mitochondrial protein frataxin. It is inherited as an autosomal recessive disease with a prevalence of 1:50.000 individuals, which makes it the most common form of inherited ataxia. Typically, onset occurs during puberty, although late and early onset cases have been described. Patients initially present with gait and limb ataxia and progressively lose their ability to walk and accomplish daily activities. Clinical manifestations also include scoliosis and skeletal deformities, dysarthria, hearing and visual impairment. Neurological manifestations are associated with degeneration of sensory neurons in the dorsal root ganglia, in the dentate nuclei of the cerebellum and posterior column of the spinal cord (Koeppen and Mazurkiewicz, 2013; Koeppen et al., 2017). Although mostly considered a disorder of the nervous system, patients also develop a hypertrophic cardiomyopathy that often leads to premature death (Tsou et al., 2011; Tiano et al., 2020). Diabetes is also present in a minority of patients (Delatycki and Corben, 2012; Cnop et al., 2013).

The underlying mutation consists of a GAA triplet repeat expansion, within the first intron of the gene coding for frataxin. The repeat expansion is homozygous in >96% of cases, while a minority of patients presents the repeat expansion on one allele and a point mutation on the other allele of the frataxin gene (*FXN*) (Galea et al., 2016). The GAA repeat expansion alters the chromatin structure and leads to epigenetic modifications that reduce the accessibility of the DNA and impair transcription of the frataxin gene, resulting in insufficient production of the corresponding protein. Longer expansions of the GAA repeats correlate with lower levels of residual frataxin, earlier age at onset and more severe disease progression (Filla et al., 1996; Montermini et al., 1997; Chutake et al., 2014; Reetz et al., 2015; Patel et al., 2016), establishing a clear link between frataxin deficiency and the pathogenesis of the disease and highlighting the therapeutic relevance of restoring frataxin levels in patients.

Frataxin is an essential mitochondrial protein (Campuzano et al., 1997; Condò et al., 2007) involved in iron metabolism and in the biosynthesis of iron-sulfur clusters (ISC; Shan et al., 2007; Martelli and Puccio, 2014; Fox et al., 2019). Its deficiency results in impaired synthesis of ISC and reduced activity of many ISC-containing mitochondrial enzymes (Rotig et al., 1997; Poburski et al., 2016), causing inefficient function of the electron transport chain, reduced ATP production and consequently overall impaired mitochondrial metabolism and defective anti-oxidant response. The inability to cope with increased oxidative stress is considered one of the most important consequences of frataxin deficiency (Gomes and Santos, 2013). These alterations at the cellular level ultimately leads to cell death, the most susceptible cells being those with high mitochondrial metabolism such as neurons, cardiomyocytes and pancreatic beta-cells (Igoillo-Esteve et al., 2015). Therefore, frataxin levels are not only crucial for cell survival but also for handling different stress responses (Condò et al., 2006, 2010; Paupe et al., 2009; Guccini et al., 2011).

Currently, there is no approved therapy to treat Friedreich ataxia. Only palliative cares are available for patients, mostly consisting of antioxidants or iron chelators, all of which, however, do not impact significantly on the neurological dysfunctions associated with the disease. Since lack of frataxin is considered the direct cause of the disease, the most straightforward therapeutic approaches are aimed at increasing frataxin protein levels. Considering that the mutation in the frataxin gene does not affect the coding sequence and the residual frataxin protein that is produced in patients is functional, it is reasonable to invest in pharmacological approaches aimed to increase the production of endogenous frataxin. Strategies to elevate endogenous frataxin levels focus on either promoting its gene expression at the transcriptional (Herman et al., 2006; Li et al., 2016; Erwin et al., 2017; Shen et al., 2020) or translational (Bon et al., 2019) level, or preventing its degradation (Rufini et al., 2011, 2015; Benini et al., 2017). Alternatively, gene therapy (Perdomini et al., 2014; Piguet et al., 2018) or protein replacement approaches (Vyas et al., 2012; Britti et al., 2018; Castro et al., 2018) are being considered by various groups, reporting encouraging results (Delatycki and Bidichandani, 2019). Pharmacological treatments under development also focus on reducing the downstream consequences of frataxin deficiency, such as mitochondrial dysfunction and oxidative stress (Zesiewicz et al., 2018a,b; Lynch and Johnson, 2021). Indeed, the inability to mount an efficient anti-oxidant defense to the increased oxidative stress observed in patients’ cells is a major cause of cellular stress and death and is considered a relevant pathogenic mechanism for Friedreich ataxia. The inefficient anti-oxidant response has been linked to a defective activation of the nuclear factor erythroid 2-related factor 2 (Nrf2) pathway (Paupe et al., 2009) and its downstream target genes, both in cells derived from FRDA patients and in FRDA animal models. One of such models, the YG8R mice exhibit reduced expression of anti-oxidant genes, such as glutaredoxin, peroxiredoxin and glutathione S-transferase (Shan et al., 2013). In line with this evidence, strategies aimed at restoring an efficient Nrf2 activation are considered promising therapeutic approaches (Petrillo et al., 2019) and several drugs have been proposed which restore appropriate Nrf2 response in FRDA cellular and animal models.

Given the urgency to identify a therapy for such a devastating disease, many research efforts have focused on drug repositioning approaches. Drug repositioning or repurposing, which consists of finding a new therapeutic indication for an already existing and approved drug, has gained considerable attention over the last decades. Conventional drug discovery process involves significant high-risk investments by pharmaceutical industry, in terms of financial resources and time to reach market approval. The cost of bringing a new drug to the market was estimated to be approximately $2 billions (DiMasi, 2018) and it may take as long as 10–12 years for a new drug to be available for patients (Matthews et al., 2016). On the other side, drug repurposing approaches can take advantage of well-established safety and toxicity profiles for already approved drugs and therefore cut down the costs associated with the development of a new drug and significantly reduce the time to the final approval, allowing patients to benefit from the availability of new therapeutics (Schcolnik-Cabrera et al., 2021). This is particularly relevant in the context of rare diseases and orphan drugs, where the reduced business opportunity makes it often not convenient for pharma companies to invest in the development of new therapies from scratch (Sardana et al., 2011).

The research for a therapy for Friedreich ataxia has indeed recently pursued repositioning strategies to accelerate the pace toward the approval of an effective therapy for patients in need. In this review we will summarize the repurposing efforts made toward the identification of approved and market-available drugs for a successful treatment of Friedreich ataxia. Compounds identified and described below can be classified as chemical drugs, biological drugs and natural products, as summarized in Table 1, and are listed following the order of their initial studies in FRDA.

**TABLE 1:** List of repurposed drugs in FRDA.

Drug	Research status	Mechanism of action in FRDA	Category
*PPARγ agonists*	Phase II placebo-controlled clinical trial (Leriglitazone, 36 patients) – Completed	Increases frataxin mRNA and protein.	Chemical drug
*Dyclonine*	Proof of concept trial in patients (8 patients) – Completed	Increases frataxin mRNA and protein. Activates Nrf2	Chemical drug
*Src inhibitors*	*In vitro* studies Patients’ cells	Increases frataxin protein.	Chemical drug
*Methylprednisolone*	Phase II open-label clinical trial (11 patients) – Completed	Unknown.	Chemical drug
*Diazoxide*	Preclinical	Increases frataxin mRNA and protein. Activates Nrf2	Chemical drug
*Dimethyl fumarate*	Preclinical	Increases frataxin mRNA and protein. Activates Nrf2. Promotes mitochondrial biogenesis.	Chemical drug
*Etravirine*	Phase II open-label clinical trial (30 patients) – Ongoing	Increases frataxin protein. No effect on frataxin mRNA levels.	Chemical drug
*Artesunate*	Phase I-II open-label clinical trial (20 patients) – Ongoing	Decreases iron overload.	Chemical drug
*Erythropoietin and derivatives*	Phase II placebo-controlled clinical trials – Completed	Increases frataxin protein. No effect on frataxin mRNA levels.	Biological drug
*Interferon-γ*	Phase III placebo-controlled clinical trial (92 patients) – Completed	Increases frataxin mRNA and protein.	Biological drug
*G-CSF*	Phase II open-label clinical trial (7 patients) – Completed	Increases frataxin mRNA and protein.	Biological drug
*Exenatide*	Phase II open-label clinical trial (16 patients) – Completed	Increases frataxin protein. No effect on frataxin mRNA levels.	Biological drug
*Nicotinamide*	Phase II open-label clinical trial (10 patients) – Completed. Double-blind, placebo-controlled phase II trial (225 patients) – Ongoing	Increases frataxin mRNA and protein.	Natural product
*NAD* + *precursor (Nicotinamide riboside)*	Phase II placebo-controlled clinical trial (72 patients) – Ongoing	Enhances mitochondrial metabolism.	Natural product
*NAD* + *precursor (MIB-626)*	Phase II open-label clinical trial (10 patients) – Ongoing	Enhances mitochondrial metabolism.	Natural product
*Acetyl-L-Carnitine*	Phase II open-label clinical trial (20 patients) – Completed	Enhances mitochondrial metabolism.	Natural product
*Resveratrol*	Phase II open-label clinical trial (27 patients) – Completed. Double-blind, placebo-controlled phase II trial (40 patients) – Ongoing	Increases frataxin mRNA and protein.	Natural product
*Thiamine*	Phase II open-label (34 patients) – Completed	Unknown.	Natural product
*Sulforaphane*	*In vitro* studies Patients’ cells	Increases frataxin mRNA and protein. Activates Nrf2.	Natural product

## Chemical Drugs

### PPARγ Agonists

PPARγ agonists are a class of drugs commonly used in the treatment of type II diabetes. They were initially proposed as a therapeutic for FRDA for their ability to activate PGC1α. The master transcriptional regulator PGC1α, which controls mitochondrial biogenesis and anti-oxidant response (Coppola et al., 2009), was indeed found to be reduced in primary fibroblasts form FRDA patients and in the KIKO mouse model (Lin et al., 2017). Initial observations on PPARγ agonists showed that the Azelaoyl-PAF increases frataxin mRNA and protein levels in fibroblasts derived from patients (Marmolino et al., 2009) and pioglitazone is able to restore anti-oxidant defense inducing the expression of SOD2 in FRDA fibroblasts and in the cerebellum of KIKO mice, through the activation of PGC1α (Coppola et al., 2009; Marmolino et al., 2010). More recently, the PPARγ agonist Leriglitazone (MIN-102, developed by Minoryx Therapeutics) was evaluated for its effect in cellular and murine models of FRDA. Leriglitazone, a metabolite a pioglitazone, currently under investigation for the treatment of X-linked adrenoleukodystrophy (Rodriguez-Pascau et al., 2021b), is an orally bioavailable drug that has been shown to have a good safety profile and, importantly, is able to cross the brain-blood barrier. Rodriguez-Pascau et al. (2021a) showed that leriglitazone promotes a 50% increase in frataxin levels in frataxin-deficient rat DRG (Rodriguez-Pascau et al., 2021a), rescues neurite morphology, mitochondrial membrane potential and prevents apoptosis. While no effects on frataxin levels were observed on frataxin-deficient rat cardiomyocytes, leriglitazone prevents lipid droplets accumulation in these cells, likely thanks to a positive effect on fatty acid beta-oxidation. The effects on the motor performances of YG8sR mice administered for 7 months with leriglitazone are, however, partial, with improvement observed only in the balance beam test (Rodriguez-Pascau et al., 2021a). A phase II, placebo-controlled, clinical trial in FRDA patient to evaluate the efficacy of this drug has been recently completed, however, the data have not been published yet^1^.

### Dyclonine

Dyclonine was proposed as a candidate therapeutic for Friedreich ataxia for its ability to increase frataxin levels and rescue downstream consequences of frataxin deficiency. In an effort to identify drugs able to restore anti-oxidant defense in FRDA cells, Sahdeo et al. (2014) used the thioredoxin oxidant diamide to induce oxidative stress and cell death in FRDA cells. Through a high-throughput screening of an FDA-approved drugs library, they identified dyclonine as a drug able to confer protection against diamide-induced oxidative stress. Dyclonine is a local oral anesthetic that has been used for more than 50 years and functions through inhibition of the sodium channels (Shelmire et al., 1955; Gargiulo et al., 1992). Dyclonine induces a significant increase in frataxin transcript and consequent increase in frataxin protein in FRDA lymphoblasts and in the cerebellum of YG8 and KIKO mouse models. Moreover, dyclonine activates the Nrf2 pathway and drives the expression of Antioxidant Responsive Elements (ARE)-containing genes. The identification of ARE elements in the frataxin locus suggests an interesting mechanistic link between the activation of Nrf2 and the upregulation of frataxin protein. However, not all Nrf2 inducers identified have been shown to upregulate frataxin (Abeti et al., 2018; Petrillo et al., 2019). Dyclonine rescues the activity of the iron-sulfur cluster (ISC)-containing enzymes aconitase and succinate dehydrogenase in FRDA lymphoblasts and YG8 mice and might provide an improvement in motor coordination in the KIKO mice. However, the dyclonine dose that induces frataxin upregulation is ∼30-fold higher than the dose required for protection against diamide, indicating that additional protective mechanisms may play a major role in protection from diamide-induced oxidative stress by dyclonine. Nevertheless, 8 FRDA patients were dosed with dyclonine oral rinse for 1 week. Six out of eight patients showed an induction of frataxin protein in cells from buccal swabs, with a trend showing a positive correlation between disease severity and responsiveness to treatment (Sahdeo et al., 2014).

### Src Inhibitors

Frataxin protein levels are controlled by the proteasome upon ubiquitination of target residue, K147, on frataxin (Rufini et al., 2011). This lysine represents a crucial site for frataxin stability since a frataxin mutant lacking K147 cannot be ubiquitinated and is more stable. Therefore, preventing ubiquitination on K147 is expected to grant frataxin an increased stability and a prolonged half-life (Rufini et al., 2011). Ubiquitination and phosphorylation are post-translational modifications that are important regulators of mitochondrial functions (Hebert-Chatelain, 2013). We have reported that frataxin is phosphorylated on Y118 by Src kinase (Cherubini et al., 2015). Interestingly, non-phosphorylatable frataxin-Y118F mutant is less ubiquitinated suggesting that phosphorylation on Y118 is required for ubiquitination. Moreover, blocking Src activity with specific inhibitors such as PP2, SU6656, Saracatinib, Bosutinib and Dasatinib increases frataxin levels. Accordingly, Src inhibitors are ineffective in human cells in which a frataxin-Y118F mutant was expressed. All the Src inhibitors can promote frataxin accumulation in a dose dependent manner, although with different efficacy. Among the Src inhibitors tested, Dasatinib appeared to be the most efficient in promoting frataxin accumulation, being still active in the nanomolar range of concentrations. Interestingly, frataxin accumulation was observed for all the different processing forms such as precursor, intermediate and mature forms. All the Src inhibitors tested could increase frataxin levels in cells overexpressing wild type frataxin, but not in cells overexpressing the non-phosphorylatable Y118F frataxin mutant, suggesting that they indeed act by inhibiting phosphorylation of Y118. In addition, these inhibitors are also effective on the levels of endogenous frataxin as shown in HEK293 cells (Cherubini et al., 2015). Dasatinib could upregulate frataxin in primary fibroblasts derived from a FRDA patient in a dose dependent manner. Furthermore, aconitase activity was also restored in FRDA lymphoblasts exposed to Dasatinib. Therefore, Src inhibitors induce frataxin expression and rescue the aconitase defect in cells derived from FRDA patients, supporting their potential therapeutic application (Cherubini et al., 2015). Src inhibitors Dasatinib and Bosutinib, reported to cross the blood-brain barrier (Porkka et al., 2008; Liang et al., 2009), have been approved for therapeutic use in humans (Hochhaus and Kantarjian, 2013; Hill et al., 2014) for the treatment of Philadelphia chromosome positive (Ph+) chronic myelogenous leukemia (CML). Further investigation should thus clarify the therapeutic potential of Src inhibitors in FRDA patients.

### Methylprednisolone

The rationale for considering corticosteroid treatment for FRDA derives from several lines of evidence. Comprehensive transcriptome analysis of PBMC derived from patients identified a number of upregulated genes associated with the innate immune response (Haugen et al., 2010; Nachun et al., 2018). Moreover, frataxin deficiency leads to steroid deficiency in flies and ovarian cells (Palandri et al., 2015). Whether inflammation contributes to the pathogenesis of FRDA or it is merely a consequence of prolonged and chronic cellular stress, has yet to be established. The observation that a FRDA patient treated with steroid for a concomitant nephrotic syndrome showed some improvement in the neurological symptoms, suggested to investigate the effects of steroid in other FRDA patients (Shinnick et al., 2016). However, an open label study on 11 FRDA patients treated with oral methylprednisolone showed that, although well tolerated, the drug induced non-significant improvement in neurological abilities except for the 1-min walk test. No changes in frataxin levels were detected in buccal cells (Patel et al., 2019).

### Diazoxide

Diazoxide, a well-known drug used to treat acute hypertension (Hutcheon and Barthalmus, 1962), was shown to increase frataxin protein levels in FRDA lymphoblastoid cell lines (Santoro et al., 2018). Diazoxide functions as a potassium channel opener that acts on the mitochondrial ATP-dependent potassium channel, resulting in vasodilation. Increase in frataxin mRNA and protein levels was observed also in the brain and the heart of YG8sR mice (Anjomani Virmouni et al., 2015). Moreover, treatment with diazoxide resulted in increase in aconitase activity and reduced protein oxidation in the brain in this mouse model (Santoro et al., 2018). The activation of the mTOR pathway, already described as an effect of diazoxide (Kwon et al., 2006), could in turn be responsible for the activation of Nrf2 (Lim et al., 2014) observed upon treatment with diazoxide in FRDA cells and could promote the anti-oxidant response. Interestingly, the mTOR pathway was also directly linked to the increase in frataxin levels following IGF-1 stimulation (Franco et al., 2012). However, results on neurological parameters in YG8sR were variable, contrasting and difficult to interpret, with some improvement on the beam walk test but overall reduced locomotor function (Santoro et al., 2018).

### Dimethyl Fumarate

Through the screening of a library of market-available drugs, Jasoliya et al. (2019) identified dimethyl fumarate (DMF) as a drug able to protect FRDA cells from cell death. DMF was previously shown to promote the survival of motor neurons and neuronal stem cells from oxidative stress-induced cell death by activating Nrf2 pathway (Wang et al., 2015; Petrillo et al., 2017). DMF is a fumaric acid derivative currently used for the treatment of multiple sclerosis and psoriasis (Venci and Gandhi, 2013) and recently proposed for the treatment of Parkinson’s disease (Lastres-Becker et al., 2016). In multiple sclerosis patients, DMF was shown to promote mitochondrial biogenesis, through the activation of Nrf2 pathway.

Treatment with DMF promotes an increase in frataxin levels in lymphoblastoid cell lines from FRDA patients and in the cerebellum of YG8 and KIKO mice (Jasoliya et al., 2019). Interestingly, an increase in frataxin mRNA had been previously observed in peripheral blood mononuclear cells (PBMC) from multiple sclerosis patients treated with DMF for 3 months. The proposed mechanism of action implies an increase in the transcription initiation at the frataxin gene and a reduction in the R-loops content. As it is also known that DMF triggers the activation of the Nrf2 pathway (Linker et al., 2011), binding of Nrf2 to putative ARE in the promoter region of frataxin could account for the increased frataxin transcription. The increased transcriptional activation of the frataxin gene could in turn reduce the formation of R-loops that is favored during transcriptional pausing of the RNA polymerase, further increasing frataxin mRNA elongation. Accordingly, cells treated with DMF present reduced R-loops frequency, which could further contribute to the resulting enhanced frataxin transcription (Jasoliya et al., 2019). DMF was also shown to promote mitochondrial biogenesis in fibroblasts derived from FRDA patients (Hayashi et al., 2017), possibly by activating the PGC-1α and Nrf1 dependent mitochondrial biogenesis pathway (Wu et al., 1999; Piantadosi et al., 2008).

More recently, the activity of DMF was tested in a *FXN* knock down model of FRDA (Hui et al., 2021). The *FXN* knock down mice represents a recently developed FRDA model, in which frataxin expression is silenced by a doxycycline-inducible frataxin siRNA. This system allows a progressive down regulation of frataxin in all the tissues, which induces a progressive and more physiological phenotype. Defects in mitochondrial biogenesis were observed in these mice following frataxin silencing (Chandran et al., 2017). Of relevance, DMF treatment rescues mitochondrial deficit caused by frataxin knock down by inducing an increase in mitochondrial copy number and restoring mitochondrial enzymes activity. Conversely, DMF orally administered to wild type mice for 2 weeks increases frataxin and cytochrome c oxidase-4 expression and aconitase activity in the brain and muscle, without affecting mitochondrial biogenesis, indicating that DMF promotes mitochondrial biogenesis only when there is a deficit caused by low frataxin expression (Hui et al., 2021).

Importantly the maximal effective dose observed in mice is equivalent or below the maximal approved dose in humans for the treatment of MS and psoriasis. Future investigations are required to assess the effect of DMF on clinical parameters in Friedreich ataxia and clinical trials in FRDA patients are currently being planned.

### Etravirine

Using a frataxin reporter system in a cell-based assay, our group performed a screening of an FDA-approved and commercially available drugs library to search for compounds able to increase frataxin amount in cells. Among a few potentially interesting candidates, our attention was focused on etravirine, given its highly favorable safety profile and its potential to be used as a chronic treatment (Croxtall, 2012; Havens et al., 2020). Etravirine (Intelence) is a non-nucleoside reverse transcriptase inhibitor (NNRTI) of the HIV enzyme (Namasivayam et al., 2019; Zhuang et al., 2020). it is currently used an anti-HIV therapy in life-long treatment regimens (Lazzarin et al., 2007; Katlama et al., 2020). Its use has been approved by FDA in patients starting from 2 years of age and above. Etravirine promotes a significant increase in frataxin levels in lymphoblasts and fibroblasts derived from FRDA patients (Alfedi et al., 2019). However, etravirine does not appear to elicit an increase in frataxin mRNA levels nor does it affect its stability. Data suggest that etravirine treatment results in an enhancement of the translation rate of frataxin, through a mechanism involving the redistribution of frataxin mRNA to the actively translating heavy polysome fraction. Interestingly, frataxin accumulation in treated patients’ cells is comparable to frataxin levels in cells from unaffected carrier sibling, suggesting a potential beneficial role of etravirine in a Friedreich ataxia therapy. Indeed, etravirine can correct some defects associated with frataxin deficiency. In lymphoblasts from FRDA patients, etravirine restores the activity of aconitase and confers protection to oxidative stress (Alfedi et al., 2019). Further studies will be required to assess whether a functional improvement can be achieved in tissues relevant for FRDA pathology, such as the heart and the nervous system. In this regard, etravirine was shown to pass into the cerebrospinal fluid of treated HIV patients, although it was found to be highly protein-bound (Nguyen et al., 2013; Havens et al., 2020).

Etravirine functions by binding to HIV reverse transcriptase (RT) in the NNRTI binding pocket and allosterically regulating its catalytic activity (Zhuang et al., 2020). However, HIV RT does not have any human counterpart and etravirine binding mode on RT is not suggestive of any obvious human target that could account for the effect on frataxin. Efforts are now focused on the identification of the relevant molecular target through which etravirine elicits frataxin accumulation. This could in turn unveil new pathways relevant for the regulation of frataxin synthesis.

Considering the excellent safety profile of etravirine and its availability as on oral medication, a phase II clinical trial to test the efficacy of etravirine in FRDA patients is currently ongoing^2^.

### Artesunate

Defects in the metabolic pathway for Transferrin receptor 1 (TfR1) palmitoylation could lead to increased cell surface expression of TfR1 (Alvarez et al., 1990). Lack of palmitoylation is also associated with increased endocytosis of transferrin-TfR1 complex and defective recycling of transferrin, leading to intracellular iron accumulation (Drecourt et al., 2018). Petit et al. (2021) have shown that fibroblasts derived from FRDA patients develop iron overload and fail to negatively regulate iron transport in response to ferric ammonium citrate. Interestingly, an increased expression of TfR1 at the plasma membrane was observed in these cells, associated with a defect in the palmitoylation pathway. Defects in the assembly of iron-sulfur cluster, observed in frataxin deficient cells, and consequent defects in the activity of ISC-containing enzymes involved in palmitate synthesis, could be responsible for the reduced palmitoylation of TfR1. This could in turn account for the increase in intracellular iron observed in FRDA patients. Artesunate is a derivative of artemisinin extracted from the artemisia plant, used for the treatment of malaria, and proposed as anti-cancer treatment for its anti-proliferative effects (Yang et al., 2021). Artesunate was shown to promote palmitoylation of TfR1 (Ba et al., 2012; Drecourt et al., 2018). Indeed, treatment of FRDA fibroblasts with artesunate restored palmitoylation levels and reduced iron overload (Petit et al., 2021). Similar effects were observed in PBMC from FRDA patients. The possibility to target TfR1 palmitoylation therapeutically in FRDA using artesunate will be explored in a clinical trial in FRDA patients^3^.

## Biological Drugs

### Erythropoietin

Erythropoietin (EPO) is a cytokine produced by the kidney that plays a major role in the regulation of erythropoiesis (Jelkmann, 1992). The initial attention toward considering EPO for FRDA came from the observation that recombinant human erythropoietin (rhuEpo) can promote frataxin increase in neuronal and cardiac cells and in PBMCs (Sturm et al., 2005) and fibroblast derived from FRDA patients, through a mechanism that does not involve an increase in frataxin mRNA transcription (Acquaviva et al., 2008). Moreover, erythropoietin exerts effects on iron metabolism, by inducing heme synthesis (Camaschella et al., 2016), and promotes mitochondrial biogenesis and oxidative respiration (Carraway et al., 2010; Plenge et al., 2012), further favoring it as a candidate therapy for FRDA. Erythropoietin is able to cross the blood-brain barrier (Brines et al., 2000), and was shown to have neuroprotective functions (Sakanaka et al., 1998; Celik et al., 2002; Bianchi et al., 2006), making it potentially attractive as a therapeutic for neurodegenerative diseases. However, a minimal portion of intravenously administered EPO can be found in the cerebrospinal fluid, suggesting that elevated doses are required to achieve significant concentration in the nervous system (Xenocostas et al., 2005).

Several clinical trials have been conducted in FRDA patients, giving different and sometimes inconsistent results. A pilot open label, 8 weeks study in 10 FRDA patients, reported increased frataxin levels, reduced expression of oxidative stress markers and improvement in neurological performances, as measured by the Scale for the Assessment and Rating of Ataxia (SARA) score (Boesch et al., 2007). Similar results were observed in a 6-months follow up study (Boesch et al., 2008) and in a dose escalation study (Nachbauer et al., 2011a), without showing, however, a clear dose-dependent correlation in frataxin accumulation. These studies highlighted also a significant drop in ferritin levels and an increase in hematocrit that required phlebotomy. Interestingly, increase in frataxin amount could be observed also in skeletal muscle biopsies (Nachbauer et al., 2011a,b, 2012). In another open-label trial, the effect of the administration of two single doses of erythropoietin at 3 months interval was assessed. This study showed long term increase in frataxin levels without altering hematocrit, but failed to show clinical benefit (Sacca et al., 2011). Another small dose-escalating placebo-controlled trial, however, failed to show significative increase in frataxin levels and had no effect on SARA scale (Mariotti et al., 2012). Similarly, inconsistent results were obtained in a larger placebo-controlled randomized trial, which highlighted, however, potential benefit in the upper limb performance (Sacca et al., 2016). Recent discoveries of an extra-mitochondrial frataxin in erythrocytes raise doubts about the possibility that the increase in frataxin promoted by erythropoietin could be ascribed to red blood cells (Guo et al., 2018).

To avoid undesired side effects due to stimulation of erythropoiesis promoted by erythropoietin, the possibility of using erythropoietin mimetics has been considered (Leist et al., 2004). Although an Epo derivative, such as carbamylated erythropoietin (CEPO), exerts similar effects on frataxin upregulation *in vitro* (Sturm et al., 2010), it does not show evidence of function in patients (Boesch et al., 2014). More recently, attention was drawn on two EPO mimetics, STS-E412 and STS-E424, which are agonists of the tissue protective EPO receptor. They were shown to promote increase in frataxin mRNA levels in human neuronal cells *in vitro* and in FRDA patients derived PBMC. Moreover, STS-E412 and STS-E424 stimulate frataxin expression in the brain of KIKO mice without exerting effect on hematopoiesis. Their ability to cross the blood brain barrier, together with their favorable safety profile and lack of hematopoiesis stimulation, encourage further studies on these compounds (Miller et al., 2017; Boesch and Indelicato, 2019).

### Interferon Gamma

Interferon gamma (IFN-γ) is a pleiotropic cytokine able to drive innate and adaptive immune responses against a variety of pathogens. A major immunological function of IFN-γ appears to involve the regulation of iron metabolism, as iron represents a key strategic asset for both bacteria and immune cells (Nairz et al., 2014). In order to prevent iron access to pathogens, in fact, IFN-γ upregulates the expression of multiple genes involved in iron storage, trafficking and redistribution between the cellular and extracellular environment (Abreu et al., 2020; Nairz and Weiss, 2020). Moreover, IFN-γ shows effects on various non-immune cell types, with direct modulation of metabolic pathways, tissue remodeling and neural cell survival (Ivashkiv, 2018). Notably, among the non-immune cells that might be affected by IFN-γ are the DRG sensory neurons, that are both capable of releasing and responding to IFN-γ, in an autocrine fashion (Neumann et al., 1997). Perhaps not surprisingly, therefore, this cytokine has been identified as potential therapeutic for the treatment of FRDA because of its ability to upregulate frataxin expression *in vitro* and *in vivo*. The treatment with IFN-γ has been shown to increase frataxin mRNA and protein levels in primary cells derived from FRDA patients (Tomassini et al., 2012). Same results were observed for frataxin protein in dorsal root ganglia (DRG) neurons from the YG8R FRDA mouse model. Importantly, FRDA mice treated with IFN-γ demonstrated enhancements of locomotor activity, improvements in motor coordination and prevention of DRG degeneration (Tomassini et al., 2012). Since IFN-γ is a drug already approved for use in other rare pediatric disorders, i.e., chronic granulomatous disease and severe malignant osteopetrosis, two different open-label pilot trials were rapidly performed. Both short-term clinical studies showed that IFN-γ was well tolerated in FRDA patients and without serious adverse effects (Seyer et al., 2015; Marcotulli et al., 2016). The first study reported a significant benefit in neurologic measures, with an improvement in the Friedreich Ataxia Rating Scale (FARS) score over 3 months of treatment (Seyer et al., 2015). Subsequently, a larger placebo-controlled phase III trial to assess the efficacy of IFN-γ in FRDA has been conducted. Unfortunately, because of underestimation of experimental variability and systematic wrong timing in clinical measure assessment, this part of the study did not allow to appreciate significant differences between the treated and placebo groups, within the 6 months of treatment. However, the 12 months open-label extension of the same study showed a potential benefit toward expected disease progression in treated patients (Lynch et al., 2019). More recently, an open-label phase II trial measuring the SARA score and using instrumental evaluations including electro/echocardiography (Vavla et al., 2020b) and Diffusion Tensor Magnetic Resonance Imaging of the brain (Vavla et al., 2020a), was completed over an observation time of 18 months. The progression of SARA was completely halted during IFN-γ treatment and slightly resumed after termination of the treatment. Significant improvements in some cardiac parameters were also observed, with a clear rebound effect after discontinuation of IFN-γ treatment. Interestingly, similar results from a case report showed reduction of cardiomyocyte damage and improvement of diastolic function after 8 months IFN-γ therapy in a patient with severe FRDA cardiomyopathy (Wyller et al., 2016). Overall, four separate clinical studies reported no critical involvement of immune responses, a satisfactory safety profile and possible disease-modifying effects of IFN-γ in FRDA patients. Frataxin measurement in blood and/or buccal cells showed heterogeneous response among patients and did not provide significant data. The results suggesting that IFN-γ treatment may slow the natural rate of FRDA progression are encouraging. However, to definitively establish the benefits of IFN-γ therapy, future clinical studies and concomitant design optimization are required.

### Granulocyte-Colony Stimulating Factor

The granulocyte-colony stimulating factor (G-CSF) is a hematopoietic cytokine promoting the proliferation and differentiation of myeloid precursors into mature granulocytes. The pharmacologic administration of this growth factor is widely used in healthy (Anderlini and Champlin, 2008) and diseased individuals (Hubel and Engert, 2003), with numerous clinical applications including allogeneic peripheral blood progenitor cell transplantation, reproductive medicine, treatment of hematopoietic and solid cancers, acute infections and myocardial infarction. The experimental administration of G-CSF, alone or in combination with stem cell factor (SCF), showed benefits toward neurological disorders in animal models (Chang et al., 2011; Song et al., 2011; Kemp et al., 2017) and has been investigated in the YG8R FRDA mice. The treatment with G-CSF or combined G-CSF/SCF revealed a transcriptional frataxin upregulation in spinal cord and cerebellum of YG8R animals, with the possible involvement of HIF-2a transcription factor (Kemp et al., 2017). Moreover, rescuing effects on aconitase activity and expression levels of Nrf2, SOD1, SOD2, catalase and glutathione peroxidase were observed. Finally, cytokine administration led to reduced neuroinflammation, reduced DRG vacuolization and significant improvements in motor coordination and locomotor activities (Kemp et al., 2017). A first open-label phase II trial to determine the effects of G-CSF on frataxin expression was recently conducted (EudraCT 2017-003084-34). The public report of this small clinical study^4^ describes interesting efficacy results concerning increased frataxin expression in PBMC and platelets of FRDA patients. G-CSF therapy showed known, mild side effects and no serious adverse events. Full results have not yet been published.

### Exenatide

In FRDA patients, frataxin deficiency causes mitochondrial dysfunction and increased oxidative stress, which lead to the apoptotic cell death of neurons and pancreatic beta cells. Loss of beta cells is the main cause of the increased prevalence of diabetes observed in FRDA patients (Cnop et al., 2012). Incretin mimetics, such as GLP-1 analogues, are currently used for the treatment and management of type 2 diabetes (Hinnen, 2017; Chia and Egan, 2020). Recently, GLP-1 analogues were shown to protect FRDA patients beta cells from apoptosis and promote survival of neurons by reducing oxidative stress (Igoillo-Esteve et al., 2020). These effects could be the consequence of restored anti-oxidant response due to the activation of Nrf2 and its downstream targets, which has been associated with GLP-1 treatment (Igoillo-Esteve et al., 2015; Fernandez-Millan et al., 2016). In addition to promoting beta cells function and improving glucose tolerance, a further study has shown that the GLP-1 analog exenatide promotes an increase in frataxin protein levels in the brain of KIKO mice. A similar effect was also observed in beta cells and neurons derived from FRDA patients iPSCs (Igoillo-Esteve et al., 2020). Along with this modest increase in frataxin amount, an increased activity of ISC-containing enzymes was also noticed, together with an overall improved mitochondrial function. Considering also its potential to cross the blood-brain barrier (Kastin and Akerstrom, 2003; Christensen et al., 2015), the protective effects of exenatide were further investigated in an open label pilot clinical trial on FRDA patients. A mild increase in frataxin levels was observed in platelets derived from patients treated for 5 weeks with exenatide. However, no improvement on neurological functions as assessed by the SARA score or Activities of Daily Living scales, was detectable. Whether this is because the period of treatment was not long enough or whether other parameters might influence the response in patients should be assessed in further studies.

## Natural Compounds

### Nicotinamide

The GAA repeat expansion in the frataxin intron 1 is known to cause epigenetic modification leading to condensed chromatin structure and reduced accessibility of the gene for transcription, resulting in reduced amount of frataxin protein. Hence, the use of HDAC inhibitors has been widely investigated for their potential ability to reverse epigenetic silencing of the frataxin gene (Herman et al., 2006; Soragni and Gottesfeld, 2016). In line with these studies, Festenstein and colleagues explored the possibility to use the class III HDAC sirtuin inhibitor nicotinamide for the treatment of Friedreich ataxia. Nicotinamide (vitamin B3) is a class III HDAC inhibitor (Avalos et al., 2005; Vaquero et al., 2007) that has been widely used as a food additive over the last years for a number of indications, without presenting significant adverse effects. Importantly, nicotinamide has the ability to cross the blood-brain barrier (Stratford and Dennis, 1994). Initial studies reported an upregulation of frataxin mRNA and protein in lymphoblastoid cell lines derived from FRDA patients, in patients PBMC and in the YG8 mouse model (Chan et al., 2013). This effect is concomitant with a reduced DNA methylation, a reduction in histone H3K9 and H3K27 methylation and an increase in histone H3 and H4 acetylation in the *FXN* gene. Importantly, nicotinamide can also restore the expression of a number of genes known to be dysregulated in frataxin deficient cells. However, other studies argue against the involvement of sirtuin in the regulation of *FXN* gene expression (Xu et al., 2009), therefore the mechanism of nicotinamide action in this context needs further evaluation. A later study, indeed, reported that while levels of H3K9 acetylation were increased in patients iPSC-derived neurons treated with nicotinamide, no significant alteration of frataxin mRNA and protein levels could be detected (Georges et al., 2019).

In an open-label, dose escalation trial in FRDA patients a significant and sustained increase in frataxin mRNA and protein amount was observed, together with a reduction in chromatin condensation markers (Libri et al., 2014). However, no effect on neurological parameters was observed in patients, possibly due to the short duration of the trial. Although nicotinamide was used at doses 200 times higher than the daily recommended allowance for its use as a vitamin supplement, it did not cause significant side effects beside nausea and vomiting. These effects could, however, hamper its use over longer regimen. Nevertheless, to assess nicotinamide long-term effects on FRDA progression through the SARA score, a multicenter, placebo-controlled, 24 months trial, using the individually determined highest tolerated doses, is about to start in Europe (Reetz et al., 2019)^5^.

### Nicotinamide Adenine Dinucleotide (NAD +) Precursors

Nicotinamide riboside (NR) is a NAD + precursor that presents numerous potential benefits for health and its use as a dietary supplement has been proposed to help in the treatment of cardiovascular, neurodegenerative, metabolic and aging-related disorders. Indeed, a natural decline in NAD + content is associated with aging (Mehmel et al., 2020). NAD + is an important cofactor in many oxidation-reduction reactions in the cell and plays a crucial role in glycolysis and oxidative phosphorylation. Accordingly, NR was shown to enhance mitochondrial function and promote oxidative metabolism (Canto et al., 2012). Moreover, the activity of the NAD + -dependent mitochondrial protein deacetylase SIRT3 plays an important role in cardiac function and stress response, and heart defects in muscle/cardiac *FXN*-KO mouse model can be rescued by inducing SIRT3 activity through NAD + precursor supplementation (Martin et al., 2017). Given the important function of NAD + in modulating mitochondrial metabolism and heart bioenergetic, supplementation with the NAD + precursor MIB-626 (Metro International Biotech, LLC) or with NR is currently being tested in FRDA patients^6,7^.

### Acetyl-L-Carnitine

Acetyl-L-Carnitine (ALCAR) is a naturally occurring compound that facilitates energy production in the mitochondria and exerts neurotrophic effects. It is proposed as beneficial in a number of conditions such as cardiovascular and neurodegenerative disorders and is currently marketed as a food supplement for the treatment of neuropathies (Maldonado et al., 2020; Pennisi et al., 2020). L-carnitine has been proposed as a therapeutic for FRDA and its effects have been tested in a placebo-controlled trial in 16 FRDA patients, showing mild but encouraging improvements in energy metabolism (Schols et al., 2005). More recently, an open-label trial with ALCAR has been performed on 20 FRDA patients, but results have not been published yet^8^.

### Resveratrol

Resveratrol is a natural product extracted from the grape, berries and nuts that has been proposed to have anti-oxidant, anti-tumor and neuroprotective properties. Although not approved for any specific indication, it is commonly used as a dietary supplement (Salehi et al., 2018). It is also characterized as an activator of the sirtuin class of HDAC (Pyo et al., 2020). Starting from the screening of a collection of FDA-approved drugs and natural compounds, Li and colleagues identified resveratrol, among others, as a compound able to elicit an increase in frataxin levels (Li et al., 2013) in cells expressing a *FXN*-EGFP genomic reporter containing a normal copy of the frataxin, as well as in lymphoblasts and fibroblasts derived from FRDA patients, both at the mRNA and protein level. Moreover, frataxin protein levels were found elevated in the brain of YG8R transgenic mice, administered with resveratrol for 3 days, suggesting the *in vivo* efficacy of this compound and indicating its ability to cross the blood-brain barrier. Nevertheless, when administered *in vitro* to neurons derived from patients iPSCs, resveratrol failed to elicit a significant increase in frataxin mRNA and protein levels (Georges et al., 2019). Similarly, a successive 3-months open label study on FRDA patients failed to detect an increase in frataxin levels. However, improvements in neurological parameters, as measured by the FARS score, and reduction in oxidative stress, as measured by reduction in the F2-isoprostanes marker, were observed in patients treated with the highest dose of 5 g/day (Yiu et al., 2015). A double-blind, placebo-controlled trial with micronized resveratrol is currently ongoing to further assess the impact of this treatment in FRDA patients^9^.

### Thiamine

Thiamine (Vitamin B1) is an important co-factor of many enzymes involved in energy metabolism within the cells, such as pyruvate dehydrogenase and alpha-keto-glutarate dehydrogenase. It also plays a role in neurotransmission, in the maintenance of mitochondrial membrane potential, glucose metabolism, oxidative stress and inflammation (Jhala et al., 2014; Xu et al., 2021). It is proposed to have a neuroprotective function. Indeed, thiamine deficiency is associated with cerebellar dysfunction (Mulholland, 2006) and neurodegenerative diseases (Jhala and Hazell, 2011). Non-coenzyme functions for thiamine have also been reported which may play a role in neuroprotection (Aleshin et al., 2019). Earlier studies reported a thiamine deficiency in the cerebrospinal fluid of FRDA patients (Pedraza and Botez, 1992; Botez and Young, 2001) and an impaired activity of pyruvate dehydrogenase (Barbeau et al., 1976; Dijkstra et al., 1984). However, the relevance of this deficiency is controversial (Bettendorff et al., 1996).

A 3-months study in two FRDA patients reported significant improvement in motor coordination and reduction in fatigue after administration of high doses of thiamine (Costantini et al., 2013). A longer open-label study in a cohort of 34 FRDA patients, which lasted between 90 and 930 days, reported a significant improvement in the SARA score and a decrease in the thickness of interventricular septum. Some patients presented an improvement in tendon reflexes and in swallowing. The effects on frataxin levels in PBMC were variable and not consistent. The treatment was well tolerated and did not present adverse effects. However, the lack of a placebo control and the fact that patients did not present a deficit in thiamine before the start of the trial, warrant careful evaluation of the effect of this vitamin in FRDA.

### Sulforaphane

Sulforaphane is an isothiocyanate derived from cruciferous plants that has recently gained considerable attention thanks to its potential anti-oxidant (Ruhee and Suzuki, 2020), antitumor (Iahtisham Ul et al., 2021), anti-inflammatory and neuroprotective effects (Calabrese and Kozumbo, 2021; Mangla et al., 2021), through the activation of the Nrf2 pathway (Petrillo et al., 2019; Uddin et al., 2020). It is currently sold as a nutritional supplement. Its activity was tested in a motor neuron cell line in which frataxin gene had been silenced (Petrillo et al., 2017). These cells present mitochondrial dysfunction, impaired anti-oxidant defense with an increased GSSG/GSH ratio and decreased Nrf2 response, and axonal degeneration (D’Oria et al., 2013; Carletti et al., 2014). Sulforaphane treatment promotes Nrf2 response and the expression of its downstream target genes, restores glutathione homeostasis and promotes axonal regeneration. Moreover, in accordance with the hypothesis linking the activation of the Nrf2 pathway with frataxin expression, an increase in frataxin levels was also observed upon treatment with sulforaphane in frataxin-silenced motor neurons. Similar results were also observed in FRDA patients derived fibroblasts (Petrillo et al., 2017). These data lend further support to the idea of targeting the Nrf2 pathway as therapeutic strategy for FRDA. Additional studies should clarify the therapeutic potential of sulforaphane in FRDA patients.

## Conclusion

Currently, there is no approved treatment to cure Friedreich ataxia or halt the progression of the disease. A number of efforts have been put into the development of an effective therapy, focusing on targeting mitochondrial dysfunction, oxidative stress and augmenting frataxin levels (Clay et al., 2019; Delatycki and Bidichandani, 2019; Gottesfeld, 2019; Zesiewicz et al., 2020). Nonetheless, drug discovery for FRDA has been challenging, for at least two reasons. First, the classical process of drug discovery implies high investments form pharmaceuticals companies and requires a very long process of development that includes medicinal chemistry-driven optimization and experimental validation before moving to the clinical phases. Second, designing clinical trials for rare neurodegenerative diseases, such as FRDA, has often proved to be tricky because of the paucity of patients and difficulties in enrolling a sufficiently large and stratified sample, and because of the heterogeneous nature of the disease with a slow and variable progression rate, which makes it challenging to identify reliable outcome measures to monitor the progression of the disease (Patel et al., 2016; Reetz et al., 2021; Rodden and Lynch, 2021; Savelieff and Feldman, 2021).

Drug repurposing is a viable alternative approach to classical drug discovery that can potentially bypass some of these hurdles (Pushpakom et al., 2019; Talevi and Bellera, 2020) and boost the discovery of a therapy for FRDA. Some of the repurposed drugs identified for FRDA could actually be turned into an available therapy for patients in the next years. By taking advantage of already established safety profiles, toxicology and pharmacokinetic studies, repurposed drugs can indeed more rapidly enter clinical trials to directly evaluate their efficacy in patients.

A repurposed drug could prove effective as it is, or could undergo some additional development and reformulation and be approved for use in a different dosage, using a different route of administration or even used in combination with other drugs. Indeed, drugs identified through repositioning screening can sometimes require optimization to increase their efficacy and achieve a therapeutically relevant effect. However, one of the most important limitation in drug repurposing approaches is that most of the times, even though the drug has been identified as able to rescue Friedreich ataxia phenotype, its relevant molecular targets in FRDA and its specific mechanism of action remain unknown. This poses a significant drawback, as lack of this knowledge significantly hinders the optimization process of the candidate therapeutic compound and the development of more efficient derivatives. On the other side, although implying a considerable research effort, target identification of potentially active compounds, identified through drug repositioning screening for FRDA, holds the possibility to unveil new molecular targets and pathways that could be relevant for understanding FRDA pathophysiology. This could add to the research field and open the way for the development of target-specific therapies for FRDA.

By integrating these efforts there is great hope that patients will benefit from a therapeutic opportunity in the near future.

## Author Contributions

AR and RT contributed to conception and designed of the study. AR wrote the first draft of the manuscript. FM and IC wrote sections of the manuscript and made substantial, direct and intellectual contribution to the work. AR, FM, IC, and RT contributed to manuscript revision. All authors read and approved the submitted version.

## Conflict of Interest

The authors declare that the research was conducted in the absence of any commercial or financial relationships that could be construed as a potential conflict of interest.

## Publisher’s Note

All claims expressed in this article are solely those of the authors and do not necessarily represent those of their affiliated organizations, or those of the publisher, the editors and the reviewers. Any product that may be evaluated in this article, or claim that may be made by its manufacturer, is not guaranteed or endorsed by the publisher.

## Footnotes

1
https://www.clinicaltrials.gov/ct2/show/NCT03917225

2
https://clinicaltrials.gov/ct2/show/NCT04273165

3
https://clinicaltrials.gov/ct2/show/NCT04921930

4
https://www.clinicaltrialsregister.eu/ctr-search/trial/2017-003084-34/results

5
https://clinicaltrials.gov/ct2/show/NCT03761511

6
https://clinicaltrials.gov/ct2/show/NCT04192136

7
https://clinicaltrials.gov/ct2/show/NCT04817111

8
http://www.clinicaltrials.gov/ct2/show/NCT01921868

9
https://clinicaltrials.gov/ct2/show/NCT03933163
